# Chitosan Grafted with Biobased 5-Hydroxymethyl-Furfural as Adsorbent for Copper and Cadmium Ions Removal

**DOI:** 10.3390/polym12051173

**Published:** 2020-05-20

**Authors:** Mariza Mone, Dimitra A. Lambropoulou, Dimitrios N. Bikiaris, George Kyzas

**Affiliations:** 1 Laboratory of Polymer Chemistry and Technology, Department of Chemistry, Aristotle University of Thessaloniki, GR-541 24 Thessaloniki, Greece; mariza@chalmers.se; 2 Laboratory of Environmental Pollution Control, Department of Chemistry, Aristotle University of Thessaloniki, GR-541 24 Thessaloniki, Greece; dlambro@chem.auth.gr; 3 Department of Chemistry, International Hellenic University, GR-654 04 Kavala, Greece

**Keywords:** chitosan, 5-hydroxymethyl-furfural, derivatives, biobased polymers, adsorption, copper, cadmium, wastewaters

## Abstract

This work investigates the application of 5-hydroxymethyl-furfural (HMF) as a grafting agent to chitosan (CS). The material produced was further modified by cross-linking. Three different derivatives were tested with molecular ratios CS/HMF of 1:1 (CS-HMF1), 2:1 (CS-HMF2) and 10:1 mol/mol (CS-HMF3)) to remove Cu^2+^ and Cd^2+^ from aqueous solutions. CS-HMF derivatives were characterized both before, and after, metal ions adsorption by using scanning electron microscopy (SEM), as well as Fourier-transform infrared (FTIR) spectroscopy thermogravimetric analysis (TGA), and X-Ray diffraction analysis (XRD). The CS-HMF derivatives were tested at pH = 5 and showed higher adsorption capacity with the increase of temperature. Also, the equilibrium data were fitted to Langmuir (best fitting) and Freundlich model, while the kinetic data to pseudo-first (best fitting) and pseudo-second order equations. The Langmuir model fitted better (higher R^2^) the equilibrium data than the Freundlich equation. By increasing the HMF grafting from 130% (CS-HMF1) to 310% (CS-HMF3), an increase of 24% (26 m/g) was observed for Cu^2+^ adsorption and 19% (20 mg/g) for Cd^2+^. By increasing from *T* = 25 to 65 °C, an increase of the adsorption capacity (metal uptake) was observed. Ten reuse cycles were successfully carried out without significant loss of adsorption ability. The reuse potential was higher of Cd^2+^, but more stable desorption reuse ability during all cycles for Cu^2+^.

## 1. Introduction

In recent years, there have been many published studies addressing the efficient decontamination or removal of various heavy metal ions from aqueous systems [1,2]. The existence of some heavy metal ions in high concentrations is a concerning issue. The latter becomes even bigger taking into consideration the allowable concentrations in aqueous systems (rivers, lakes, sea, etc.). Finding an appropriate way to manage and treat wastewaters containing those toxic metal ions is pertinent. Researchers applied various different methods to achieve efficient treatment, to address some important issues as [3], including; (i) the feasibility of the process, (ii) the environmental-friendly approach [4], (iii) the simplicity and low cost potential [3], and (iv) the efficiency of the removal, etc. [1,2]. There is not one factor of the aforementioned that is more important compared to other. Finding a method that satisfies the combination of all issues as described above is of crucial importance. Recently, significant attention has been paid to adsorption technique [5,6,7,8,9,10,11,12,13,14,15,16,17,18,19,20], which are easily applied to the final stages of wastewater treatment plants (WWTPs), with the aim of removing all residues (ions) that were not separated and removed from the previous stages.

Adsorption is believed to be a good candidate as an applied technique because it is very simple, can be achieved in a short time-frame (from some minutes to less hours), the instrumentation is cheap (adsorptive columns with majorly plexiglas), and the whole operation requires few personnel. Another point of interest is in selecting the most appropriate adsorbent material to apply. For this reason, numerous studies were published reporting a huge quantity of adsorbent materials, which were used in various WWTPs. Materials from agricultural wastes were used in their raw form for adsorption or as precursors for further modification (activated carbons). Moreover, nanomaterials are very “hot” nowadays for adsorption application, but their major drawback is the complexity of their synthesis, which exponentially increases their final cost. On the other hand, some good candidates are polymeric materials that can be easily modified, in order to obtain additional functional groups (grafting reactions). The latter is crucial because this method can drastically improve the adsorption capacity of the material. Furthermore, polymeric adsorbents have the advantage of being transformed to an extremely rigid solid adsorbents (after suitable cross-linking reactions), which allows the reusability potential (cyclic economy). Chitosan is a promising polymer that has been extensively studied by many researchers and our team. Chitosan is an amino-based polysaccharide and has a chemical type poly-β-(1→4)-2-amino-2-deoxy-*D*-glucose. This polymer can be produced in abundance by following *N*-deacetylation reaction of chitin, which is its origin compound [21,22,23]. Chitin has chemical type poly-β-(1→4)-*N*-acetyl-*D*-glucosamine and widely is believed to be an abundant natural biopolymer [24,25]. Chitin exists in crustaceans, cartilages of molluscs, cuticles of insects and cell walls of micro-organisms. Chitosan is a promising material because it includes some important physical properties as macromolecular structure, the non-toxic potential, the biocompatible nature of the polymer, biodegradable properties in line with the low-cost due to its origin [22]. Apart from this, it can be used to numerous applications, including pharmacology, chemical technology and biotechnology, cosmetic industry, industries using membranes, food market and industry, adsorption technology, etc. [26,27,28,29,30,31,32,33,34,35,36,37,38]).

Another important topic for consideration is identifying a heavy metal ion target for removal. Some studies focus on the decontamination (focus on metal ions) following the order of toxicity; the latter means that they investigate the removal majorly of the most toxic metal ions (Hg, Cd, etc.) [39,40,41,42]. Other researchers target on highly-concentrated metal ions (Cu, Zn, etc.) [43,44] independent on their toxicity. It must be mentioned that it is utopic to believe that there is one technique, or more specifically, one adsorbent material, which can be successfully remove all heavy metal ions. For this reason, in the present study, we select to remove with chitosan adsorbent materials one very toxic (Cd) and one highly-concentrated metal ion (Cu) from aqueous media. 

The next step is to find a good agent that will be grafted onto the chitosan backbone in order to give better properties to the final product. Our team has already synthesized a chitosan derivative which was grafted with 5-hydroxymethylfurfural (HMF) [45]. HMF is a biobased furan compound which is the product of dehydration [46]. 

The primary target of the current study is the application of the grafted derivatives CS-HMF to remove Cu^2+^ and Cd^2+^ from aqueous solutions. Three different derivatives were tested based on preliminary findings [45]; (i) the first derivative was synthesized by using 1 mol CS and 1 mol HMF, meaning 1:1 ratio and abbreviated as CS-HMF1; (ii) the second derivative was synthesized by using 1 mol CS and 0.5 mol HMF, meaning 2:1 ratio and abbreviated as CS-HMF2; (iii) the third derivative was synthesized by using 1 mol CS and 0.1 mol HMF, meaning 10:1 ratio and abbreviated as CS-HMF3. For this purpose, a complete experimental design was set up. CS-HMF was characterized both before and after metal ions adsorption by using scanning electron microscopy (SEM), as well as Fourier-transform infrared (FTIR) spectroscopy, thermogravimetric analysis (TGA), and X-Ray diffraction analysis (XRD). The adsorption evaluation of the chitosan derivatives was multi-parametric, because the effect of (i) pH, (ii) initial ion concentration, (iii) temperature on adsorption was studied. Also, various eluants were tested as desorption agents and the reuse potential was examined. Langmuir and Freundlich equations were applied to fit the experimental equilibrium data, while the pseudo-first (PS1) and pseudo-second (PS2) order equations were applied to model the experimental kinetic data.

## 2. Materials and Methods 

### 2.1. Materials

All reagents were purchased by Sigma-Aldrich (Berlin. Germany). Chitosan (CS) in powder form was purchased for the synthesis of chitosan derivatives. It was selected the high molecular weight form (310–375 kDa, >75% deacetylated) and 5-hydroxymethylfurfural (HMF) as grafting agent. Glutaraldehyde (50% v/v) and sodium tripolyphosphate (98%) were purchased to cross-link the chitosan chains. Some other reagents that were used for the main synthesis of the material (NaCl, Na_2_HPO_4_, KCl, K_2_HPO_4_·3H_2_O and HCl) were also supplied from the same company. To prepare the heavy metal ion stock solutions, copper(II) sulfate pentahydrate (CuSO_4_·5H_2_O (≥98%)) and cadmium nitrate tetrahydrate (Cd(NO_3_)_2_·4H_2_O ((98%)) were purchased from Sigma-Aldrich. Moreover, many question why HMF was not tested as an adsorbent. However, the functional groups of HMF, which can act as adsorptive sites, are one hydroxyl and one carbonyl group. On the other hand, the functional groups of chitosan are namino groups, n hydroxyl groups. So the chitosan (polymer) is by far more appropriate adsorbent material. Also, some preliminary experiments with the use of HMF as adsorbent were carried out, but too many stability problems existed due to the extreme pH-conditions.

#### Synthesis of CS-HMF Derivatives

**Figure 1:**
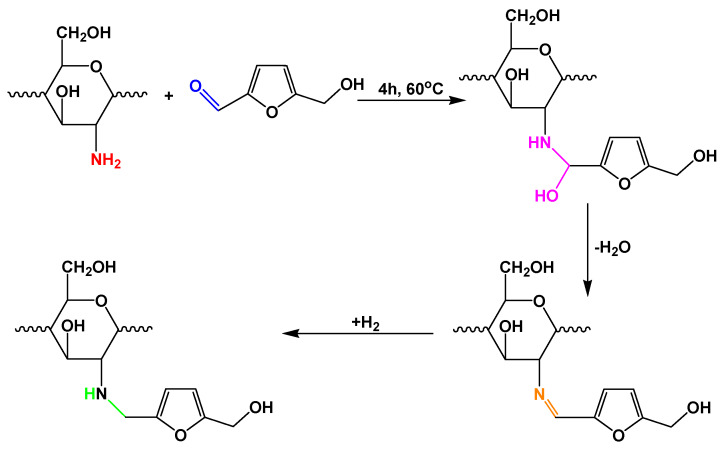
Synthesis route of CS-HMF.

The synthesis of CS-HMF derivatives was described in our previous study [45]. Briefly, CS-HMF derivatives were synthesized using three different molecular ratios of 1:1, 2:1 and 10:1 mol/mol (CS/HMF) named as CS-HMF1, CS-HMF2, CS-HMF3, respectively. Briefly, the preparation route of CS-HMF1 was based on the following procedure. A total of 1 g of CS was dissolved in 2% (v/v) acetic acid and stirred overnight. Afterwards, 0.37 g of HMF were dissolved in 5 mL of ethanol and then added in CS solution. The solutions were placed in a two-neck round-bottom flask. The reaction was carried out at 60 °C for 4 h using magnetic stirring. NABH_4_ was added drop wised during the reaction. The resulting product was lyophilized and purified by extraction with acetone in Soxhlet apparatus for 24 h. A similar procedure was followed for CS-HMF2, CS-HMF3. For the preparation of cross-linked CS-HMF derivatives, which were used only for swelling studies, 1 g of CS-HMF was dissolved in 50 mL of 4% (v/v) aqueous acetic acid solution. 1% (v/v) of GLA solution was added to 1% (w/w) TPP (pH 6) solution. The chitosan solution was then poured into this coagulant solution for gelation. This solution was stirred overnight and then lyophilized to take the final derivatives (Figure 1).

### 2.2. Characterization Techniques

The characterization of the prepared grafted chitosans was based on four basic techniques. FTIR was used to examine the functional groups of chitosans after synthesis (before adsorption experiments). The same characterizations were applied after heavy metal ions experiments to examine the interaction between ions and chitosan groups. The FTIR spectra were taken using a Perkin-Elmer FTIR spectrometer (model Spectrum One, Perkin Elmer, Dresden, Germany). Infrared (IR) absorbance spectra were taken in the range of 450–4000 cm^−1^ at 4 cm^−1^ resolution with 20 co-added scans. All spectra submitted to baseline correction and normalization to 1. The patterns of XRD were taken using Rigaku MiniFlex II diffractometer with Bragg-Brentano geometry (θ, 2θ) and a Ni-filtered CuKα radiation. Analysis was performed on the synthesized chitosans. The samples were scanned over the internal range of 5°–60°, step 0.05°, rate 1° min^−1^. SEM images were performed with electron microscope (model Zeiss Supra 55 VP, Jena, Germany). The accelerating voltage was 15 kV and the scanning was performed *in-situ* on a sample powder. Thermal stability of the samples was measured (6 mg of sample was used for each measurement) by TGA using TGA thermal analyzer (model Pyris 1 TGA, Perkin-Elmer, Dresden, Germany) with the following settings: (i) 10 K/min as heating rate, and (ii) 100 mL/min as flow rate of nitrogen atmosphere.

### 2.3. Adsorption/desorption Experiments

#### 2.3.1. Adsorption Experiments

The adsorption experimental design is dived into 3 main subsections, investigating the effect of some important parameters. For all adsorption experiments, before running each experimental series separately, stock aqueous solutions of copper or cadmium ions (1000 mg/L) were prepared in volumetric flasks by weighting the appropriate mass of salt (CuSO_4_·5H_2_O or Cd(NO_3_)_2_·4H_2_O) and filling with deionized water. It must be noted that adsorption was carried out by agitation of conical flasks which are placed in thermostatically-controlled shaking bath (model Grant Instruments OLS Aqua Pro, Cambridge, UK). After the end of each experiment, the residual ion concentration was found with atomic absorption spectroscopy (AAS) (Perkin–Elmer Analyst 400 composed of FIAS 100 Flow Injection System).

##### Effect of pH

To carry out the pH-effect adsorption experiments, *C*_0_ = 100 mg/L of ion solution (Cu^2+^ or Cd^2+^) was used as initial ion concentration in *V* = 20 mL of deionized water. The agitation rate was adjusted at *N* = 160 rpm (*T* = 25 °C) and the process lasted for *t* = 24 h. The mass of chitosan derivatives added was *m* = 0.02 g per flask, while the adjustment of pH in solution was achieved with micro-additions of HCl (0.01 mol/L) or NaOH (0.01 mol/L). The residual ion concentrations (*C_e_*) in the liquid phase (after the end of adsorption) were measured with AAS and the removal of ions was determined as (Equation (1)):

(1) $$R=\frac{\left(C_{0}-C_{e}\right)}{C_{0}}\cdot 100\%$$

##### Effect of Contact Time

To carry out the kinetic adsorption experiments, *C*_0_ = 120 mg/L of ion solution (Cu^2+^ or Cd^2+^) was used as initial ion concentration in *V* = 20 mL of deionized water. The agitation rate was adjusted at *N* = 160 rpm (*T* = 25 °C). The mass of chitosan derivatives added was *m* = 0.02 g per flask, while the adjustment of pH was at 5, because it was found to be the optimum after pH-effect adsorption experiments in solution was achieved with micro-additions of HCl (0.01 mol/L) or NaOH (0.01 mol/L). At fixed time-intervals (*t* = 5–1440 min), the residual ion concentration was analyzed. The residual ion concentrations (*C_e_*) in the liquid phase were also calculated as described in Section “Effect of pH”. To fit the experimental data, two widely-used kinetic equations were used; (i) pseudo-first order kinetic equation [47] (Equation (2)), (ii) and pseudo-second order kinetic equation [48,49,50] (Equation (3)),

(2) $$C_{t}=C_{0}-(C_{0}-C_{e})\left(1-e^{-k_{1}t}\right)$$

(3) $$C_{t}=C_{0}-(C_{0}-C_{e})\left(1-\frac{1}{1+k_{2}t}\right)$$

where *C_t_* is the ion concentration at time *t*, *k*_1_ and *k*_2_ are the kinetic constants of pseudo-first, and pseudo-second order kinetic equations, respectively. 

##### Effect of Initial Ion Concentration and Temperature

To carry out the equilibrium adsorption experiments, *C*_0_ = 10–300 mg/L of ion solution (Cu^2+^ or Cd^2+^) was used as initial ion concentration in *V* = 20 mL of deionized water. The agitation rate was adjusted at *N* = 160 rpm (*T* = 25, 45, 65 °C). The mass of chitosan derivatives added was *m* = 0.02 g per flask, while the adjustment of pH was at 5, because it was found to be the optimum after pH-effect adsorption experiments. The duration of the process/contact time (*t*) was the optimum found from kinetic experiments. The following equation is used to calculate the equilibrium amount in the solid particle (*Q_e_*) (Equation (4)):

(4) $$Q_{e}=\frac{\left(C_{0}-C_{e}\right)V}{m}.$$

Langmuir (Equation (5)) [51] and Freundlich (Equation (6)) [52] isotherm equations were applied to simulate the experimental equilibrium data,

(5) $$Q_{e}=\frac{Q_{m}K_{L}C_{e}}{1+K_{L}C_{e}}$$

(6) $$Q_{e}=K_{F}C_{e}{}^{1/n}$$

where *Q_m_* (mg/g) is the theoretical maximum adsorption capacity; *K_L_* (L/mg) is the Langmuir equilibrium constant; *K_F_* (mg^1−1/*n*^ L^1/*n*^ g^−1^) is the Freundlich constant; *n* (dimensionless) is the Freundlich constant.

#### 2.3.2. Desorption and Reuse Experiments

To set-up desorption experimental design, primarily adsorption experiments were carried out using the optimum conditions found. In the present study, *m* = 0.02 g, *V* = 20 mL, *C*_0_ = 120 mg/L, *T* = 25 C, pH = 5. Then, the chitosan derivatives were separated from the adsorbate solution with filtration (pore-sized membranes). The metal-loaded derivatives (those after metal ion adsorption) were then placed to conical flasks which have 20 mL of different eluants. The eluants were deionized water adjusted at pH = 2, 3, 4, 5, and 6. Then the desorption percentage was estimated.

## 3. Results and Discussion

The discussion of experimental findings starts with the presentation of characterization results (SEM, XRD). FTIR analysis will be analyzed in the section of “pH-effect adsorption experiments” so as to export a proposed adsorption mechanism. Then, the adsorption evaluation is presented based on isotherms and kinetics. The reuse potential is confirmed with the evaluation of desorption and reuse experiments.

### 3.1. Evaluation of Characterization Techniques: SEM, XRD, TGA and FTIR

**Figure 2:**
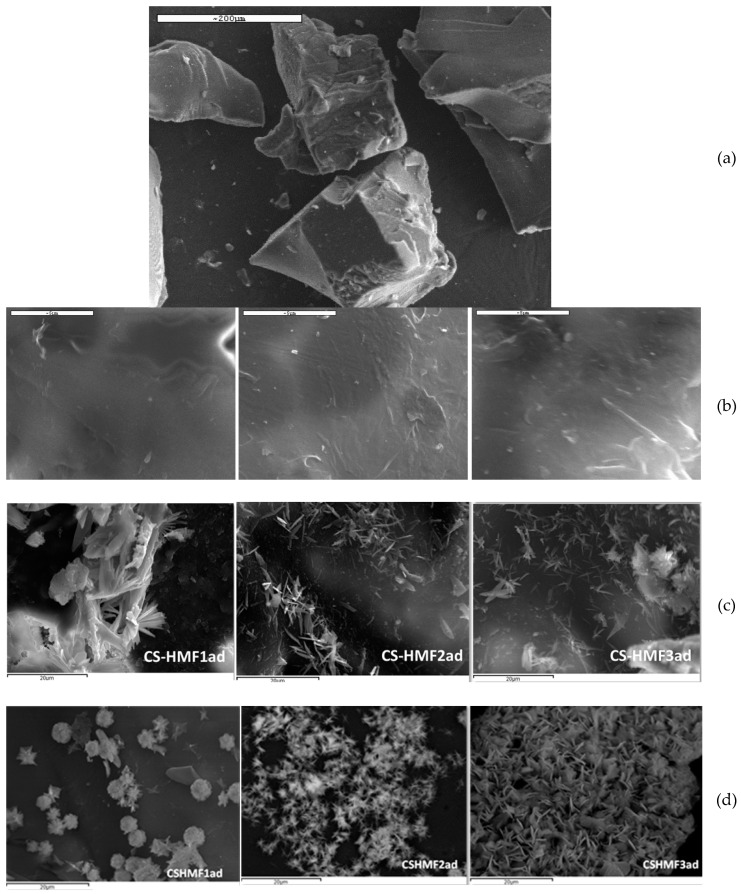
SEM images: (**a**) CS0; (**b**) CS-HMF derivatives before adsorption; (**c**) CS-HMF derivatives after Cu^2+^ adsorption; (**d**) CS-HMF derivatives after Cd^2+^ adsorption.

An important technique for evaluating the morphology of the material is scanning electron microscopy. Figure 1 demonstrates the SEM images of the chitosan derivatives. To compare the morphology, images were also taken after metal ion adsorption. All images (before and after adsorption) demonstrated the same morphology. The surface is not smooth, but with irregular-shape, harsh and scrabbly. This morphology was probably the result of modifying reactions (cross-linking and grafting), which is obvious comparing Figure 2a where the smooth surface of neat chitosan is completely different than the surface of final products (Figure 2b,c). Also, it was found that the materials before adsorption had a slightly smoother surface than those after metal adsorption. This is due to the surface interaction among various groups (functional) of the derivatives and metal ions. Also, the grinding of the chitosan particles after during synthesis, as well as the different degree of grafting caused some shape irregularities (Figure 2b,c).

**Figure 3:**
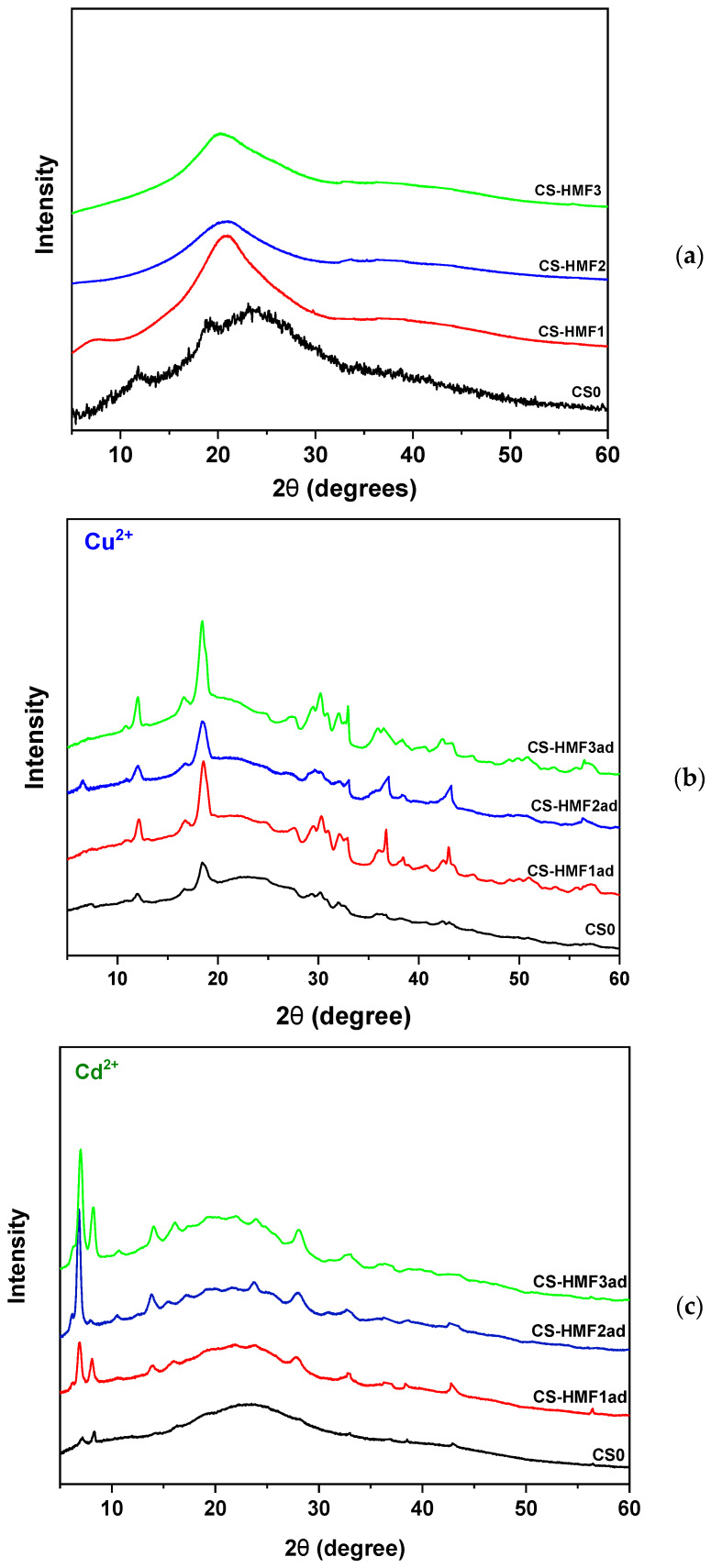
XRD patterns of the synthesized chitosan derivatives; (**a**) before adsorption; (**b**) after Cu^2+^ adsorption; (**c**) after Cd^2+^ adsorption.

Figure 3 presents the XRD diffractograms of neat chitosan and chitosan derivatives prepared before, and after, metal ions adsorption. Neat chitosan displayed the classic semi-crystalline nature structure, which can be confirmed from the two broad peaks at 2θ = 11° and 20°, which clearly confirmed the polymer [53] (Figure 3a). XRD patterns of the derivatives suggest complete amorphous structure; this can be verified by the one and only broad peak at ~20°. The grafting of HMF on chitosan caused the reduction of its folding ability, suggesting crystal structure formation. All patterns of metal-loaded CS-HMF showed amorphous structure. This is due to the adsorption mechanism, which is a combination of chelation and electrostastic interactions. The most interesting is that this was same in both copper and cadmium ion adsorption. Indicatively, the XRD patterns for the case of Cu^2+^-loaded derivatives are given in Figure 2b.

**Figure 4:**
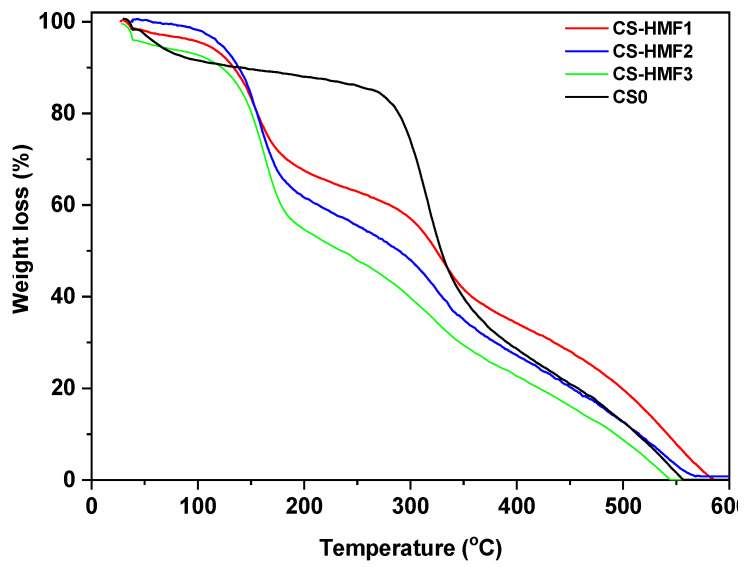
TGA analysis of the metal-loaded CS-HMF derivatives.

Figure 4 displays the TGA curves of the prepared chitosan derivatives. In the case of CS0, the first weight loss step (5%–8%) up to 120 °C is attributed to the loss of water. At temperature higher than 250 °C, the weight loss is about 40–45 wt % (this can be characterized as the major decomposition step). This corresponded to the decomposition of chitosan with reactions as dehydration of the saccharide rings and depolymerisation [54]. The thermal decomposition of derivatives presented similar behavior. There are four degradation steps. There is approximately 4% weight loss at 116 °C due to the loss of some water molecules (moisture). From 150 to 200 °C a second weight loss step is observed (37%) due to the removal of oxygen-based functional groups of the derivatives. The gradual weight loss at >250 °C is related to further degradation/removal of functional groups. The complete decomposition ends at 590 °C. Also, it can be stated that the increase in HMF grafting results in a decrease in thermal stability and increasing weight loss, compared to the neat chitosan. This is obvious from Figure 4, especially at temperature over 250 °C, where the difference of weight loss among the derivatives is about 5%–10%.

### 3.2. Adsorption Evaluation

#### 3.2.1. Effect of pH

**Figure 5:**
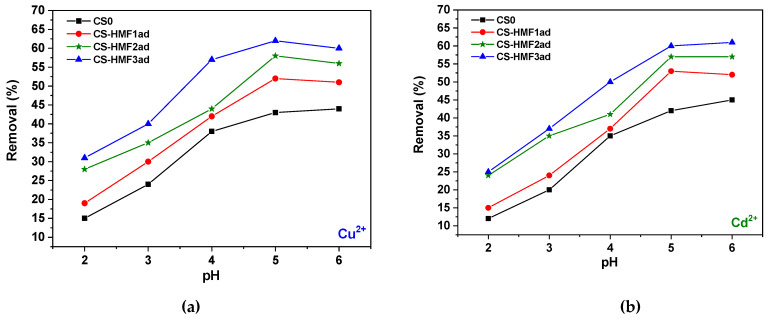
Effect of pH on the removal of (a) Cu^2+^ and (b) Cd^2+^ by CSO, CSO-HMF1ad, CSO-HMF2ad, CSO-HMF3ad. Experimental conditions: *C*_0_ = 100 mg/L, *V* = 20 mL, *N* = 160 rpm, *T* = 25 °C, *t* = 24 h, *m* = 0.02 g.

It is known that the effect of pH on adsorption plays important role on the success of the process. Figure 5 presents the pH-behavior of the process in the range of 2–6. As be observed for Figure 5, the trend of the pH-curves is the same for all derivatives both in Cu^2+^ and Cd^2+^ adsorption. The highest removal percentage was found at pH = 6 in all cases. Comparing the efficiency of the derivatives, CS-HMF3 material (310% grafting) showed the maximum removal over the whole pH-range. It is known that the metal ions adsorption majorly follows the bridge mode. This model suggest the binding of two (or more) amines from one chitosan chain with the metal cation (copper o cadmium in the present work) [43]. Other published studies suggest another model (pendant) by which the interaction takes place between one amine of chitosan (as pendant) and the cation [43]. It also important to mention that the majority of studies refer that the highest removal percentage in the case of Cu^2+^ and Cd^2+^ adsorption is in the range of 4–6 [1,2,55].

At low pH values (high acidic), two types of interactions might occur: (i) Electrostatic repulsion between positive metal ions and positively charged (protonated) amino groups of chitosan (those which have not reacted with cross−linker) (Equation (7)), and (ii) deposition/diffusion of metal ions onto chitosan network (Equation (8)):

(7) $${\mathrm{Cu}}^{2+}\;\mathrm{or}\;{\mathrm{Cd}}^{2+}\;\cdot \;\cdot \;\cdot \;\cdot \;\cdot \;\cdot \;\cdot \;\cdot \;\cdot \;\cdot \mathrm{repulsion}\;(\mathrm{high}\;\mathrm{acidic}\;\mathrm{pH})\;\cdot \;\cdot \;\cdot \;\cdot \;\cdot \;\cdot \;\cdot \;\cdot \;\cdot \;\cdot \mathrm{CS}-NH_{3}^{+}$$

Explaining the above consideration, at low pH values (high acidic), the amino groups of chitosan were protonated (NH_3_^+^), reducing the number of binding sites available for metal ions uptake (i.e., uncharged NH_2_ groups for chelation). Therefore, metal ions (Cd^2+^ or Cu^2+^) had to compete with H^+^ for adsorption sites on adsorbent’s surface. However, the grafting of HMF added carbonyl and hydroxyl groups onto the chitosan matrix. In water, carbonyl groups (−C=O) are found in equilibrium as:

(8) $$CS-C=O\;\overset{}{\underset{}{⇄}}CS-{C=O}^{-}{+H}^{+}$$

The latter in chitosan can be found only at pH > pKa (6.9), but here these values were not studied. As the pH increased from high (2.0 < pH < 4.0) to less acidic regions (4.0 < pH < 6.0), the major adsorption interaction was the chelation/complexation between amino or hydroxyl groups of chitosan (those which remained unreacted after the cross−linking) and the positive metal ions (Equation (9)):


Cu^2+^ or Cd^2+^ · · · · · · · · · ·chelation (low acidic pH) · · · · · · · · · ·CS − NH_2_ or CS − OH. (9)

Explaining the mechanism of chelation, the main sites at these pH−conditions were the amino and hydroxyl groups of chitosan, given the nitrogen of the amino group and the oxygen of the hydroxyl group had a pair of electrons, which could add themselves to a proton or a cation by coordinated covalent bonds [56]. The attraction of the electron pair by the atom’s nucleus was stronger in the oxygen. While, on the other hand, nitrogen had a greater tendency to donate its pair of electrons to a metal ion to form a complex through a coordinated covalent bond [57]. According to the major adsorption mechanism proposed (Figure 7), metal ions (Cu^2+^ or Cd^2+^) with empty orbitals functioned as a Lewis acid capable of accepting electron pairs. In contrast, the amino and hydroxyl groups without non−shared electron pairs functioned as Lewis bases, donating their pair of electrons. This behavior depended on the solution pH [56]. 

Generally, the adsorption mechanism is crucial to further understand the process of heavy metal ion removal onto different adsorbents, but identifying the adsorption mechanism of heavy metal ions by chitosan is very tedious and complicated [55]. Until now, some researchers have made some progress in the areas. In general, the overwhelming majority of the heavy metal ions are adsorbed to the surface of modified chitosan derivatives (MCS) through different interactions including electrostatic attraction, chemical bonding (viz. complexation and chelation), ion exchange, and Van der Waals force. However, functional groups of MCS can significantly influence these interactions which are characterized by different methods (such as FTIR, XPS, SEM-EDX, and XRD). In acidic solutions, the adsorption mechanism of other heavy metal cations (such as Cu^2+^, Cd^2+^, Pb^2+^, Hg^2+^ and Zn^2+^) onto chitsan is chemical bonding. The lone pair of electrons present in O, N and S of the respective hydroxyl, amino and thiol groups present in the MCS can be supplied to the empty atomic orbital of M^2+^, which makes the MCS–metal complex form in the surface of the adsorbent. In an earlier study, Cd(II) adsorption onto cross-linked chitosan/PVA beads was explained by complexation, which interacted through the electron pair sharing between Cd^2+^ and N and O atoms of the functional groups (i.e., amine and hydroxyl) in chitosan/PVA [58]. In a similar study an adsorption mechanism for the binding of Pb(II), Cd(II), and Cu(II) to Ca(II)–CS microspheres was also explained by complexation [59]. Based on the XPS spectra, the authors suggested that a lone pair of electrons in the nitrogen atom was donated to the shared bond between the N atom and metals, resulting in a decrease in electron cloud density of the nitrogen atom and an increase in binding energy. Recently, the adsorption mechanism of Pb(II) and Cd(II) on poly(itaconic acid)-grafted chitosan, with different cross-linking methods, were suggested: (i) Chelation between nitrogen atoms of amino groups of chitosan (those that remained unreacted after the cross-linking) and metal ions and (ii) electrostatic attraction between carboxylate ions of chitosan (those from grafting) and positive metal ions [42]. The attraction of the electron pair to the atom’s nucleus was stronger in oxygen, and nitrogen had a greater tendency to donate its pair of electrons to a metal ion to form a complex through a coordinated covalent bond.

**Figure 6:**
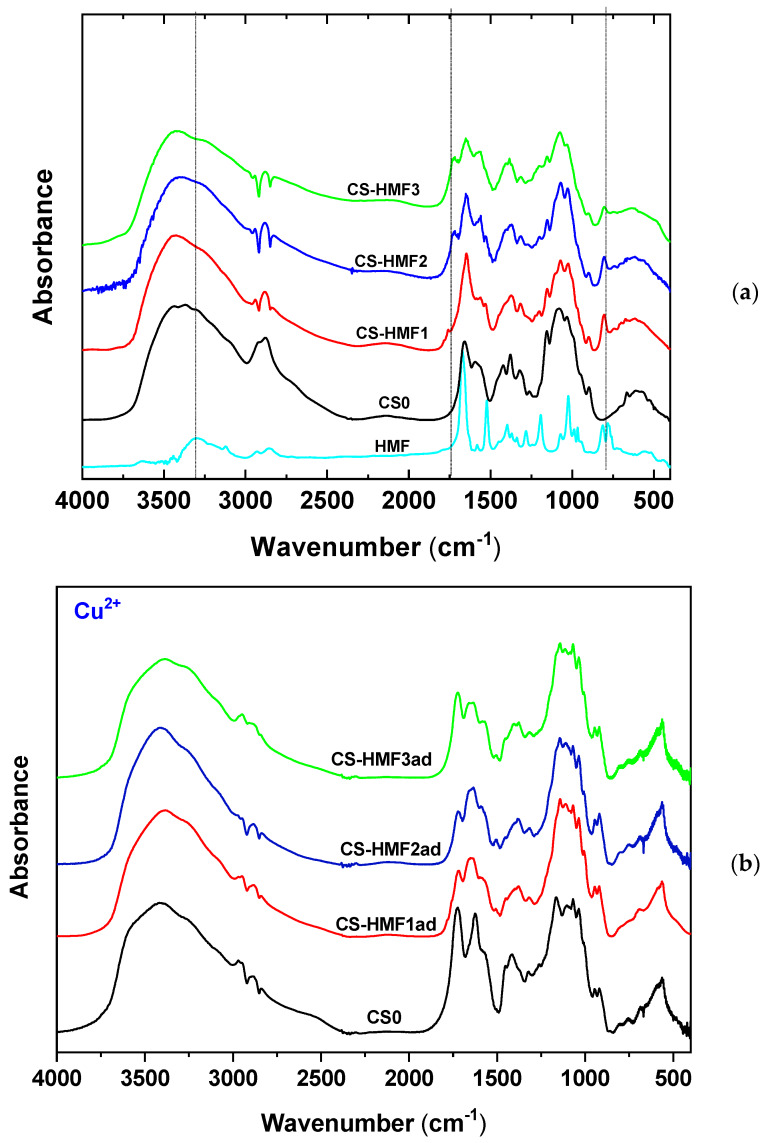

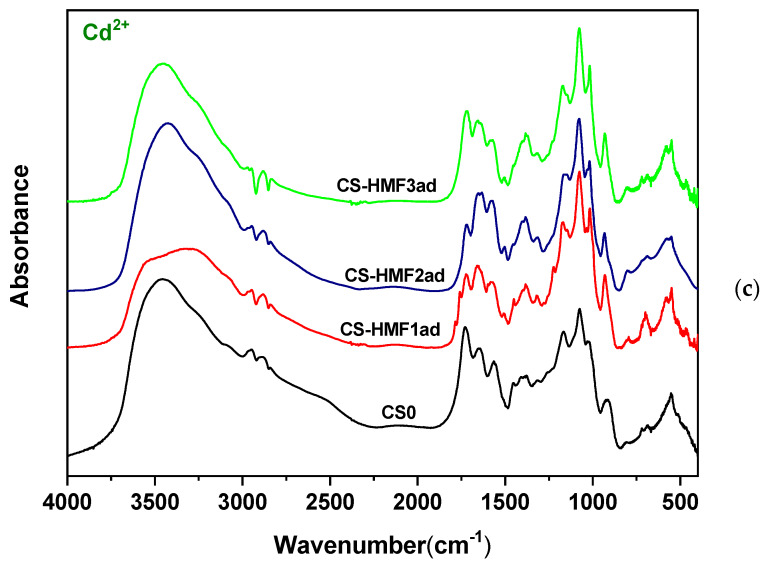
FTIR spectra of the synthesized chitosan derivatives; (**a**) before adsorption; (**b**) after Cu^2+^ adsorption; (**c**) after Cd^2+^ adsorption.

For the reasons explained above, the removal of ions is small at highly acidic conditions both for Cu^2+^ adsorption (CS0, 15%; CS-HMF1ad, 19%; CS-HMF2ad, 28%; CS-HMF3ad, 31%) and for Cd^2+^ adsorption (CS0, 12%; CS-HMF1ad, 15%; CS-HMF2ad, 24%; CS-HMF3ad, 25%). As the pH of solution increased, the deprotonation of the chitosan hydroxylic groups begin to deprotonate, therefore an electrostatic-like interaction may exist among Cu^2+^ or Cd^2+^ and deprotonated hydroxylic groups of chitosan. The latter can be also confirmed with FTIR spectroscopy as illustrated in Figure 6. 

**Figure 7:**
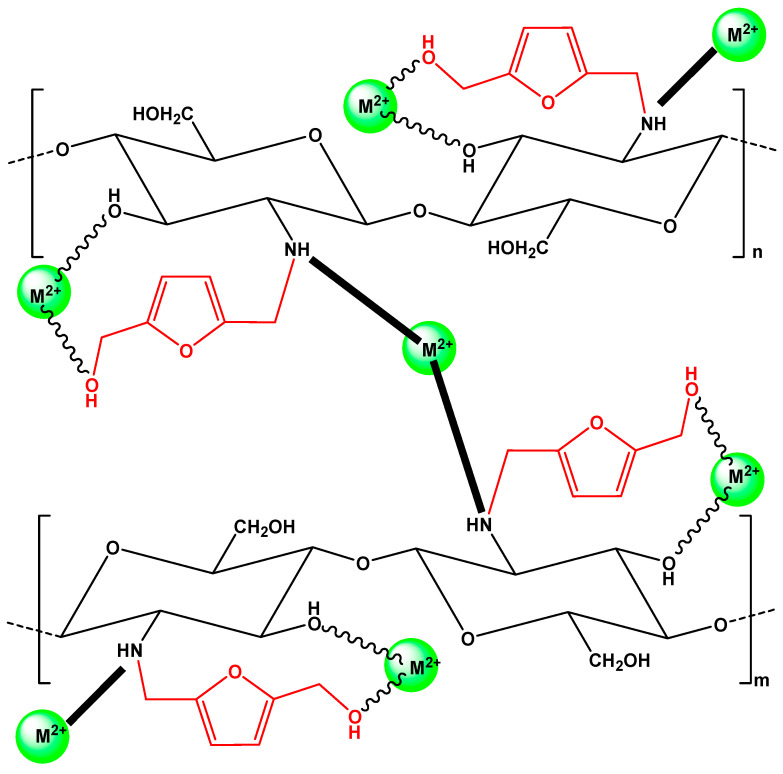
Proposed adsorption mechanism.

According to these spectra, the interaction of metal ions with hydroxyl groups can be confirmed by the shift band of hydroxyl groups after adsorption in all derivatives. Especially, in CS-HMF1ad from 3463 to 3427 cm^−1^, in CS-HMF2ad from 3438 to 3401 cm^−1^, in CS-HMF3ad from 3418 to 3425 cm^−1^. Also to avoid precipitation phenomena (pH > 6 where Cu(OH)_2_ and Cd(OH)_2_ are formed), the optimum pH value selected for further adsorption evaluation was pH = 5. For example, cadmium metal majorly can be existed as Cd^2+^ at pH values lower than 8, while and from this value up to pH = 9, Cd(OH)^+^ ions are created. At pH > 9, Cd(OH)_2_ begins to form and at pH > 13 the Cd(OH)_3_^−^ anions predominate [60]. At pH = 5, the removal for Cu^2+^ was: CS0, 43%; CS-HMF1ad, 52%; CS-HMF2ad, 58%; CS-HMF3ad, 62%. Similarly, the removal for Cd^2+^ was: CS0, 42%; CS-HMF1ad, 53%; CS-HMF2ad, 57%; CS-HMF3ad, 60%. The proposed adsorption mechanism is schematically illustrated in Figure 7. 

#### 3.2.2. Effect of Contact time

**Figure 8:**
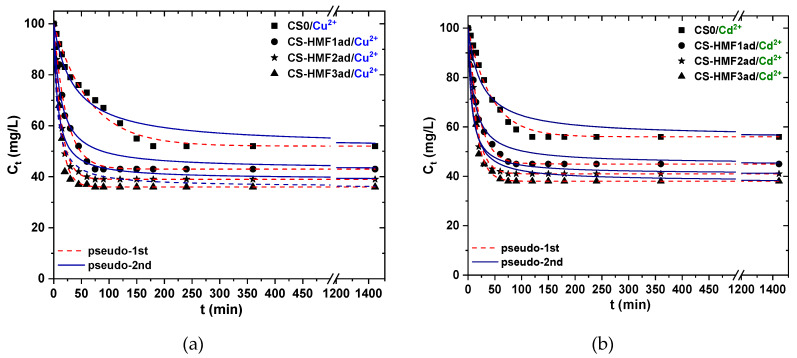
Kinetic data for the removal of (**a**) Cu^2+^ and (**b**) Cd^2+^ by CS0, CS-HMF1ad, CS-HMF2ad, CS-HMF3ad. Fitting to PS1 and PS2 equations. Experimental conditions: *C*_0_ = 100 mg/L, *V* = 20 mL, *N* = 160 rpm, *T* = 25 °C, ph = 5, *m* = 0.02 g.

Next to pH-effect experiments, another crucial factor is the contact time effect. Figure 8 shows this kinetic trend. Both metal ions acted in the same manner regarding the effect of contact time. CS0 has different kinetic behavior than the derivatives. Specifically, the first time period (5–180 min) the Cu^2+^ removal is gradual, while then a plateau was observed. In the case of Cd^2+^, the aforementioned time period is shorter (5–120 min). 

On the other hand, CS-HMF derivatives present a clear two-steps process. At first, a sharp decrease in the ion concentration is observed from 5 to 75 min and then the equilibrium (plateau) was reached. The different kinetic behavior among CS0 and CS-HMF derivatives can be attributed to the grafted functional groups, which favour the whole process. The experimental kinetic data were fitted to pseudo-1st and pseudo-2nd order kinetic equations. The relative parameters were calculated (Table 1). Pseudo-1st order equation fitted better (for both metal ions) the experimental data based on the correlation coefficients calculated (Cu^2+^: 0.983 < R^2^_ps1_ < 0.993; Cd^2+^: 0.985 < R^2^_ps1_ < 0.996).

#### 3.2.3. Effect of Initial Ion Concentration and Temperature

**Figure 9:**
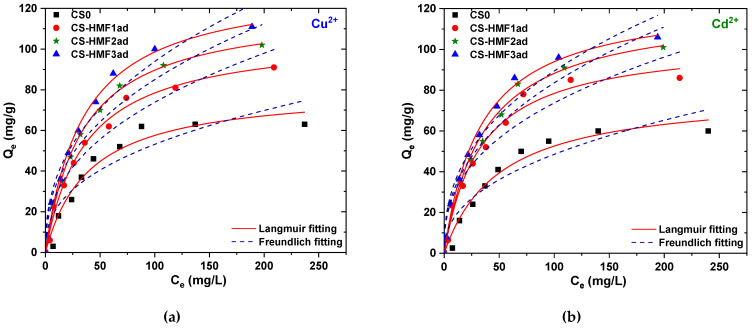
Equilibrium data (*T* = 25 °C) for the removal (**a**) Cu^2+^ and (**b**) Cd^2+^ by CS0, CS-HMF1ad, CS-HMF2ad, CS-HMF3ad. Fitting to Langmuir and Freundlich equations. Experimental conditions: *C*_0_ = 10–300 mg/L, *V* = 20 mL, *N* = 160 rpm, *T* = 25 °C, *t* = 200 min, pH = 5, *m* = 0.02 g.

Figure 9 shows the experimental equilibrium data as well as the fitting curve of the isotherms curves of CS-HMF derivatives in the case of metal ions adsorption (copper and cadmium ions). Also, all isothermal parameters resulted from the fitting of experimental data (*T* = 25 °C) to the Langmuir and Freundlich equations are reported in Table 2.

Given that this process has not a pre-determined value of adsorption capacity, the upper limit of the adsorbed amount of ions is expressed by using the maximum theoretical adsorption capacity (*Q_m_* in Table 2). The resulted correlation coefficients (R^2^) obtained for the Langmuir model were not as high (0.959 ≤ R_L_^2^ ≤ 0.999) as those of Freundlich (0.858 ≤ R_F_^2^ ≤ 0.959). CS-HMF3ad derivatives (310% grafting) presented higher adsorption capacities (25 °C) both, for Cu^2+^ (*Q_m_* = 133 mg/g) and Cd^2+^ (*Q_m_* = 125 mg/g) than those of CS-HMF2ad derivatives (230% grafting) (Cu^2+^, *Q_m_* = 120 mg/g; Cu^2+^, *Q_m_* = 119 mg/g) and CS-HMF1ad derivatives (130% grafting) (Cu^2+^, *Q_m_* = 107 mg/g; Cu^2+^, *Q_m_* = 105 mg/g). So, the different grafting influenced the adsorption capacity of the samples versus the adsorption of the same metal. Table 2 shows that by increasing the grafting from 130% to 310%, an increase of 24% (26 mg/g) was observed for Cu^2+^ adsorption and 19% (20 mg/g) for Cd^2+^.

**Figure 10:**
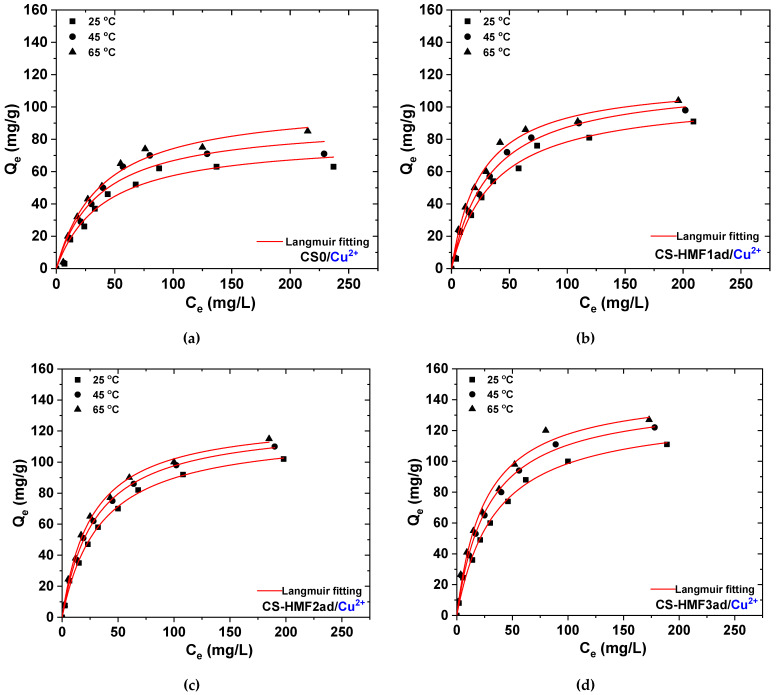
Isotherms for the removal of Cu^2+^: (**a**) CSO; (**b**) CSO-HMF1ad; (**c**) CSO-HMF2ad; (**d**) CSO-HMF3ad. Experimental conditions: *C*_0_ = 10-300 mg/L, *V* = 20 mL, *N* = 160 rpm, *T* = 25, 45, 65 °C, *t* = 200 min, pH = 5, *m* = 0.02 g.

The temperature-effect was also studied for 45 and 65 °C. As it was found, the best fit was achieved with the Langmuir equation, and the equilibrium data for 45 and 65 °C were fitted only to Langmuir equation (Table 2). All derivatives presented the same behavior by increasing from *T* = 25 to 65 °C, an increase of the adsorption capacity (metal uptake) was observed. For Cu^2+^ adsorption (Figure 10), CS-HMF1ad enhanced its capacity from 107 mg/g at 25 °C to 117 mg/g at 45 °C, and finally 118 mg/g at 65 °C. CS-HMF2ad also demonstrated similar findings (from 120 mg/g at 25 °C to 126 mg/g at 45 °C, and finally 129 mg/g at 65 °C); CS-HMF3ad also improved its capacity by increasing the temperature (from 133 mg/g at 25 °C to 142 mg/g at 45 °C, and finally 147 mg/g at 65 °C). The above increase was approximately 9% for CS-HMF1ad, 7.5% for CS-HMF2ad and 11% for CS-HMF3ad.

**Figure 11:**
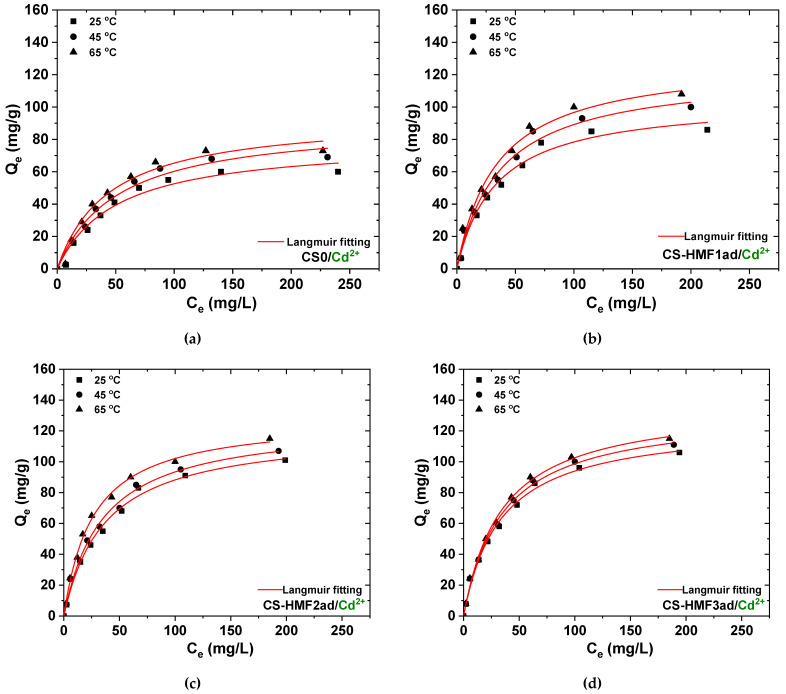
Isotherms for the removal of Cd^2+^: (**a**) CSO; (**b**) CSO-HMF1ad; (**c**) CSO-HMF2ad; (**d**) CSO-HMF3ad. Experimental conditions: *C*_0_ = 10-300 mg/L, *V* = 20 mL, *N* = 160 rpm, *T* = 25, 45, 65 °C, *t* = 200 min, pH = 5, *m* = 0.02 g.

In the case of Cd^2+^ adsorption (Figure 11), CS-HMF1ad enhanced its Q_m_ from 105 mg/g at 25 °C to 122 mg/g at 45 °C, and finally 130 mg/g at 65 °C. Similar adsorption behavior was revealed for CS-HMF2ad (from 119 mg/g at 25 °C to 126 mg/g at 45 °C, and finally 129 mg/g at 65 °C) and CS-HMF3ad (from 125 mg/g at 25 °C to 132 mg/g at 45 °C, and finally 138 mg/g at 65 °C). The above increase was approximately 24% for CS-HMF1ad, 8% for CS-HMF2ad and 10% for CS-HMF3ad.

### 3.3. Desorption/Reuse Cycles

**Figure 12:**
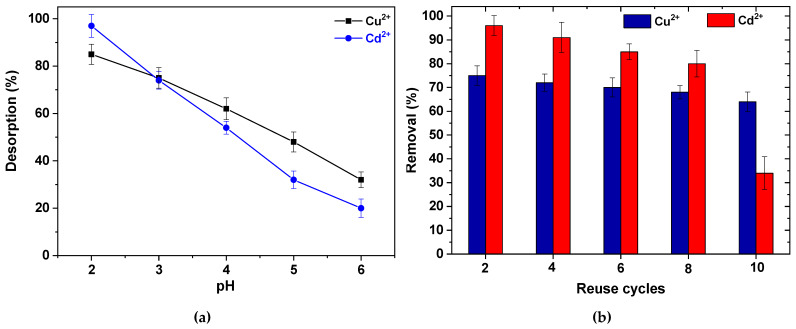
(**a**) Effect of pH on desorption of Cu^2+^ and Cd^2+^ from CS-HMF3 derivative. (**b**) Reuse cycles of CS-HMF3. Experimental conditions (adsorption): *C*_0_ = 100 mg/L, *V* = 20 mL, *N* = 160 rpm, *T* = 25 °C, *t* = 200 min, pH = 5, *m* = 0.02 g; Experimental conditions (desorption): *V* = 20 mL, *N* = 160 rpm, *T* = 25 °C, *t* = 24 h, pH = 2, *m* = 0.02 g. The figures present the experimental points with errors bars calculated after standard deviation (n = 4).

It is very important to determine the reuse potential of a prepared adsorbent. For this reason, before running reuse cycles, the optimum desorption conditions (eluant) must be found. It must be noted that the desorption and reuse experiments were carried out with only the best adsorbent (CS-HMF3) found after the evaluation achieved in previous section. In Figure 12a, the desorption percentage of CS-HMF3 is illustrated in both metal ions. It is noted that the highest desorption ability was found at pH = 2 (97% for cadmium and 86% for copper). This can be attributed to the the fact that the chelated bonds between metal ions and amino (or hydroxyl) groups of chitosan were weakened decreasing the pH of solution, so the desorption presented higher percentages at acidic pH values [61]. However, given the adsorption process is not considered to be fully reversible, incomplete desorption can be suspected for all pH values.

The reuse cycles of CS-HMF derivative revealed the reuse potential of the material (Figure 12b). In the case of Cu^2+^ adsorption and desorption from CS-HMF3, the loss in the removal ability until the 2nd cycle is 3% (from 75 to 72%), while proceeding to the next stages the loss until the tenth cycle was totally 11%. The latter can be characterized as very promising finding given the gradual damage of the material. On the other hand, in the case of Cd^2+^ adsorption and desorption, the loss in the removal ability until the 2nd cycle is 5% (from 96 to 91%), while proceeding to the next stages the loss until the 10th cycle was totally 62% (from 96 to 34%). Although the initial desorption was very high, cadmium ions removal did not happened easily increasing the reuse cycles. 

The need of regeneration must be highlighted, because without regeneration, pollutants may be released into the environment by disposal or storage of spent adsorbents. Moreover, storage and dumping of spent adsorbents may lead to explosions, fires, and stinks [62,63]. Therefore, the spent adsorbent should be stabilized before disposal. The reuse process is performed by repeating the adsorption and desorption cycles, providing substantial economic and environmental benefits [63,64]. Indeed, researchers are more inclined toward regeneration and reuse of adsorbents because of the high cost of production, stabilization, and disposal. The cost of adsorbent preparation enhances the importance of regeneration. An adsorbent can be applied industrially if it can be used in several regeneration processes (adsorption-desorption cycle). Regeneration of adsorbent generally leads to the recovery of adsorbate molecules, reuse of adsorbent in the adsorption process, reduced secondary waste and cost of adsorption process, and also helps in the understanding of the adsorption process mechanism [55].

Aqueous eluants (water with inorganic acids as HCl or HNO_3_) are the most widely used acidic desorption agents for desorption of heavy metal ions from chitosan and its derivatives. Desorption by acidic eluents is more effective than that of other eluents. In acidic environments, the high number of H^+^ ions in the solution desorbs adsorbed ions. Compared with heavy metal ions, H^+^ ions have stronger affinity to adsorb on chitosan functional groups and higher diffusivity coefficient because of their smaller radii [65]. Consequently, the cationic exchange between H^+^ and adsorbates, the protonation of adsorption sites, and the replacement of H^+^ ions with heavy metal ions in adsorbent–adsorbate complexes release adsorbed ions into the desorption solution and reduce the heavy metal ions that bind on adsorbents [63,66]. This reaction is:


Chitosan-NH_2_-Metal + H^+^→Chitosan-NH_3_^+^ + Metal.

The supply of high number of H^+^ ions can weaken the interaction between adsorption groups and metal ions. Cl^−^ from HCl can easily form a complex with heavy metal ions and then release to the solution [67]. Wan et al. [66] evaluated the desorption of Cu(II) and Pb(II) ions from chitosan coated sand in a batch system using tap water (pH 7) and diluted HCl solutions (pH 1 and 3). The results signified that more metal ions could be recovered under acidic conditions (Cu(II) (99.22%) and Pb(II) (97.91%) at pH 1). Yan et al. [68] also applied acidic solution at different pH levels (1.0 to 12.0) for regeneration of glutaraldehyde (GLA) cross-linked carboxymethylated chitosan beads loaded with Cu(II) ions. Desorption efficiencies were approximately zero at higher pHs (pH > 5.0) and approximately 99% at pH 1. This phenomenon could be due to the relatively weak interactions between metal ions and functional groups on the adsorbents at low pH values, whereas a higher stability of the formed bond is observed between adsorbate and adsorbent under neutral conditions. During the regeneration experiment, increasing the number of adsorption-desorption cycles leads to decrease the adsorption and desorption efficiencies.

Despite the aforementioned advantages, a major drawback is that the use of acidic eluent will generate “acidic waste”. For this reason, some ideas are given as: (i) Optimization to find the minimum volume of eluant, which is required to obtain the maximum desorption ability, (ii) combination of thermal degradation in line with acidic eluants, to avoid using a large quantity or volume of acidic solutions, etc. After the discharge of this volume of acidic waste which is minimized, the latter can be more easily managed and treated due to the smaller volume and lower concentration of heavy metals. In any case, all these limitations are outweighed by all the advantages described in the previous paragraph.

## 4. Conclusions

This work investigates the application of 5-hydroxymethyl-furfural (HMF) as grafting agent to chitosan (CS). The material produced was further modified by cross-linking. Three different derivatives were tested with molecular ratios CS/HMF of 1:1 (CS-HMF1), 2:1 (CS-HMF2) and 10:1 mol/mol (CS-HMF3)) to remove Cu^2+^ and Cd^2+^ from aqueous solutions. The highest removal for both metal ions was at pH=5. The Langmuir model fitted better (higher R^2^) the equilibrium data than the Freundlich equation. The PS1 equation fitted better (higher R^2^) the experimental kinetic data than the PS2 equation. By increasing the HMF grafting from 130% (CS-HMF1) to 310% (CS-HMF3), an increase of 24% (26 m/g) was observed for Cu^2+^ adsorption and 19% (20 m/g) for Cd^2+^. By increasing from T=25 to 65 °C, an increase of the adsorption capacity (metal uptake) was observed. 10 reuse cycles were successfully carried out without significant loss of adsorption ability. Higher the reuse potential of Cd^2+^, but more stable desorption reuse ability during all cycles for Cu^2+^.

## Figures and Tables

**Table 1:** Kinetic parameters for Cu^2+^ ad Cd^2+^ adsorption onto chitosan-HMF derivatives (fitting to PS1 and PS2 kinetic equations).

	Cu^2+^				Cd^2+^			
	Pseudo-1^st^ Order		Pseudo-2^nd^ Order		Pseudo-1^st^ Order		PSEUDO-2^ND^ ORDER	
	*K*_1_	*R* ^2^	*K*_2_	*R* ^2^	*K*_1_	*R* ^2^	*K*_2_	*R* ^2^
Adsorbent	(min^−1^)	(-)	(g mg^−1^ min^−1^)	(-)	(min^−1^)	(-)	(g mg^−1^ min^−1^)	(-)
CS0	0.01551	0.983	0.02789	0.952	0.02315	0.991	0.04075	0.918
CS-HMF1ad	0.04337	0.993	0.08099	0.931	0.04947	0.996	0.09361	0.951
CS-HMF2ad	0.07260	0.991	0.14141	0.930	0.06594	0.985	0.15812	0.893
CS-HMF3ad	0.08171	0.986	0.16296	0.919	0.06806	0.993	0.13228	0.936

**Table 2:** Equilibrium parameters for Cu^2+^ ad Cd^2+^ adsorption onto CS-HMF derivatives.

		Cu^2+^						Cd^2+^					
		Langmuir Equation			Freundlich Equation			Langmuir Equation			Freundlich Equation		
	*T*	*Q_m_*	*K_L_*	*R* ^2^	*K_F_*	*n*	*R* ^2^	*Q_m_*	*K_L_*	*R* ^2^	*K_F_*	*n*	*R* ^2^
Adsorbent	(°C)	(mg/g)	(L/mg)	(-)	mg^1−1/n^ L^1/n^ g^−1^	(-)	(-)	(mg/g)	(L/mg)	(-)	mg^1−1/n^ L^1/n^ g^−1^	(-)	(-)
CS0	25	81	0.025	0.959	8.697	2.543	0.858	79	0.019	0.968	6.787	2.341	0.879
	35	91	0.027	0.951				91	0.020	0.971			
	45	103	0.026	0.977				94	0.022	0.971			
CS-HMF1ad	25	107	0.027	0.991	11.258	2.449	0.944	105	0.030	0.980	12.560	2.602	0.920
	35	117	0.030	0.992				122	0.027	0.984			
	45	118	0.039	0.991				130	0.029	0.985			
CS-HMF2ad	25	120	0.029	0.997	13.226	2.475	0.956	119	0.027	0.990	12.664	2.440	0.956
	35	126	0.034	0.999				126	0.029	0.993			
	45	129	0.038	0.997				129	0.038	0.997			
CS-HMF3ad	25	133	0.029	0.995	14.292	2.446	0.959	125	0.030	0.995	14.053	2.489	0.957
	35	142	0.036	0.993				132	0.029	0.995			
	45	147	0.040	0.987				138	0.029	0.996			
